# Association between Health Literacy and Prevalence of Obesity, Arterial Hypertension, and Diabetes Mellitus

**DOI:** 10.3390/ijerph19159002

**Published:** 2022-07-24

**Authors:** Božica Lovrić, Harolt Placento, Nikolina Farčić, Metka Lipič Baligač, Štefica Mikšić, Marin Mamić, Tihomir Jovanović, Hrvoje Vidić, Sandra Karabatić, Sabina Cviljević, Lada Zibar, Ivan Vukoja, Ivana Barać

**Affiliations:** 1 General County Hospital Požega, 34 000 Požega, Croatia; marinmamic@hotmail.com (M.M.); ihi.pakrac@gmail.com (T.J.); hrvoje.vidic@gmail.com (H.V.); sabina.cviljevic@gmail.com (S.C.); ivan.vukoja@pozeska-bolnica.hr (I.V.); 2 Faculty of Medicine, Josip Juraj Strossmayer University of Osijek, 31 000 Osijek, Croatia; harolt.placento@obnasice.hr (H.P.); metkalipicbaligac@gmail.com (M.L.B.); ladazibar@gmail.com (L.Z.); 3 Nursing Institute “Professor Radivoje Radić”, Faculty of Dental Medicine and Health Osijek, Josip Juraj Strossmayer University of Osijek, 31 000 Osijek, Croatia; smiksic@fdmz.hr (Š.M.); ibarac@fdmz.hr (I.B.); 4 General Hospital Našice, 31 500 Našice, Croatia; 5 Department of Surgery, University Hospital Centre Osijek, 31 000 Osijek, Croatia; 6 General County Hospital Murska Sobota, 9 001 Rakičan, Slovenia; 7 Alma Mater Europaea, 2 000 Maribor, Slovenia; 8 General Hospital Pakrac and Hospital of Croatian Veterans, 34 550 Pakrac, Croatia; 9 Department of Respiratory Diseases “Jordanovac”, University Hospital Center, 10 000 Zagreb, Croatia; skarabat@kbc-zagreb.hr; 10 Department of Health Care, University of Applied Health Sciences, 10 000 Zagreb, Croatia; 11 Faculty of Health Studies, University in Mostar, 88 000 Mostar, Bosnia and Herzegovina; 12 Department for Nephrology, Clinical Hospital Merkur, 10 000 Zagreb, Croatia; 13 Faculty of Medicine, University of Rijeka, 51 000 Rijeka, Croatia

**Keywords:** arterial hypertension, obesity, diabetes mellitus, health literacy

## Abstract

Background: Health literacy (HL) is linked to many health outcomes, including self-management of chronic diseases. The aim of this study was to assess the association of health literacy with the prevalence of obesity, arterial hypertension (AH), and type 2 diabetes mellitus (T2DM). Methods: This cross-sectional, single-center study included 500 patients (42.2% male and 57.8% females; median age, 63 years (interquartile range, 42–73)) hospitalized at General County Hospital in Požega, Croatia, between July and October 2020. The Short Assessment of Health Literacy for Croatian Adults (SAHLCA-50) questionnaire was used. Descriptive statistics (median with interquartile range (IQR), frequency, and percentages) and binary logistic regression were utilized. Results: Patients with AH had an inadequate level of health literacy as compared to those without AH (32 vs. 40 points; Mann–Whitney U test, *p* < 0.001). Patients with T2DM scored 31 points versus 39 points in patients without T2DM (Mann–Whitney U test, *p* < 0.001). Patients suffering from both AH and T2DM scored 31 points versus 33 points in those with either AH or T2DM and 41 points in patients without AH and T2DM (Kruskal–Wallis test, *p* < 0.001). There were no statistically significant differences in SAHLCA-50 scores according to the patient body mass index. Conclusions: An inadequate level of health literacy is significantly associated with AH and T2DM but not with obesity. Male gender, low level of education, rural place of residence, retirement, and older age are significant predictors of inadequate health literacy.

## 1. Introduction

It is estimated that approximately 41 million people die from noncommunicable diseases, accounting for 71% of all-cause deaths worldwide [1]. Cardiovascular diseases are responsible for the majority of deaths from noncommunicable diseases, with 17.9 million deaths per year, followed by malignant diseases, respiratory diseases, and diabetes mellitus (DM), with 9.3, 4.1, and 1.5 million deaths per year, respectively. These four disease groups account for more than 80% of premature deaths [1].

World Health Organization (WHO) data show that 47,600 people died from noncommunicable diseases in Croatia in 2020, yielding a 92% mortality rate of these diseases. Unhealthy dietary habits and lack of physical exercise can result in arterial hypertension (AH), elevated blood glucose, elevated lipids, and obesity [2]. Arterial hypertension is the leading metabolic risk factor for premature death at the global level, accounting for 19% of deaths worldwide [3], accompanied by overweight/obesity and elevated blood glucose [4,5]. Metabolic syndrome refers to a group of pathological conditions that includes central obesity, arterial hypertension, and carbohydrate and lipid metabolism disorders, which increases the risk of developing type 2 diabetes, cardiovascular diseases, and obesity energy intake [6]. Obesity cannot be viewed as a simple phenomenon; instead, it is a complex concept of cumulative imbalance of energy intake and consumption in the context of complex interactions of environmental factors that influence behavior by modulating energy intake and consumption [7] with significant influence of genetic and developmental factors. It was observed that among obese patients, there are metabolically healthy obese patients; however, patients who have metabolic disorders in addition to obesity belong to the metabolically unhealthy obese patient group [8].

Research shows that metabolically unhealthy obese people have a more pronounced parameter of oxidative stress, which plays a significant role in the development of cardiovascular diseases [9], whereas the use of antioxidants is associated with a reduction in the level of markers of oxidative stress [10,11]. In the recent years, scientists have made considerable efforts to better understand the pathophysiology of metabolic syndrome, as well as its components and complications. Nevertheless, further research is required because some aspects of the above-mentioned problems remain unknown, and many other factors can affect health.

The worldwide prevalence of AH in the adult population aged >20 years has been estimated as 26.4% and is expected to increase to 60% by 2025 due to economic development and demographic patterns [12]. The European Health Interview Survey (EHIS) implemented in Croatia in 2014–2015 revealed that 26.8% of females and 22.3% of males reported elevated arterial blood pressure in the previous year [13]. Accordingly, AH has been recognized as a major public health issue at the global level [14].

DM is a serious, even alarming public health problem, considering that currently, even half a billion people suffer from T2DM worldwide, with an increasing tendency, expected to increase to 700 million people by 2045 [15].

According to Croatian National Diabetes Registry data, T2DM was diagnosed in 315,298 adult individuals in 2019, showing a rising tendency. However, studies carried out to date indicate that only 60% of T2DM-affected people have been diagnosed with the disease; therefore, the total number of T2DM patients is estimated to be more than 500,000 adult individuals [16].

Obesity is the main cause of a number of associated diseases, such as cardiovascular diseases, cancer, T2DM, and other disorders that can lead to morbidity and mortality [17], making it the fifth leading risk factor for mortality worldwide [18,19,20]. The latest estimates show that 30–70% and 10–30% of the adult population in the European Union (EU) member countries suffer from overweight and obesity, respectively [21]. In 2019, normal body weight was recorded in only 34% of the adult population in Croatia, placing Croatia at the very top of European countries according to the proportion (65%) of overweight and obese population [22]. 

Studies have pointed to the association between health behavior [23,24,25,26] and adverse health outcomes, such asT2DM, AH, and cancer [27,28,29]. Health literacy, along with beliefs and attitudes, affects the perception of treatments and their effects on health. Due to insufficient knowledge and understanding of the disease and its symptoms, patients do not undertake the necessary self-care activities [30]. Additionally, an inadequate level of health literacy was associated with low education level and low socioeconomic status of patients [31].

The European Health Literacy Survey revealed 12% of the European population to have inadequate and 35% limited health literacy [32]. A study on health literacy in Croatia among adolescents showed an adequate level of health literacy in 49% of adolescents, whereas significant differences were observed with regard to the school they attend [33]. A study conducted on the general population revealed that 58% of the population has an inadequate level of health literacy [34]. These results have instigated interest in such research in order to assess the level of health literacy and its association with the prevalence of obesity, AH, and T2DM in hospitalized patients.

The aim of the present study was to assess the association between health literacy and the prevalence of obesity, AH, and T2DM.

## 2. Materials and Methods

The study was conducted at the General County Hospital in Požega, Croatia, from July to October 2020. All study patients signed an informed consent form for participation in the study. Inclusion criteria were as follows: hospitalized patients aged ≥ 18 years, conscious, speaking Croatian language, free from cognitive damage, literate, and willing to take part in the study. As the study took place during the COVID-19 pandemic, patients suspected of or infected with COVID-19 were excluded. Sample size was not calculated a priori, but all patients hospitalized during the 4-month study period and meeting the above-mentioned criteria were included. There were a total of 500 study patients (42.2% males and 57.8% females; median age, 63 years (interquartile range, 42–73)).

A survey questionnaire was designed to collect patient data on the year of birth, gender, education, employment, and place of residence. Data on body weight, body height, and diagnosis of AH and T2DM were retrieved from patient medical records. Nutritional status was assessed by the standard body mass index (BMI) according to the WHO criteria as follows: BMI < 18.5 underweight; BMI 20–24, normal weight; BMI 25–30, overweight; and BMI > 30, obesity.

The instrument used in the study was the Short Assessment of Health Literacy for Croatian Adults (SAHLCA-50) questionnaire [35], the Croatian version of the Linguistic Adaptation of the Short Assessment of Health Literacy for Spanish Adults (SAHLSA-50) [36]. The questionnaire was language-adapted and validated in the Croatian language [37] and submitted to psychometric analysis [35]. The questionnaire consists of fifty terms (core words). Study patients used 4 × 5 cm plastic cards. A medical term was written in bold on the top of the card, and two words, i.e., the key word and a distractor, were written at the bottom. The examiner asked the patients to read the upper word aloud; then, the examiner read the two words written below and asked the subject to tell which of the two was more similar to the upper word. If the patients did not know which word was more similar, he/she answered, “I don’t know”. It should be noted that there was no room for guessing. The examiner had a form into which he discretely noted the answers. If considered necessary, the examiner repeatedly explained the instructions during the interview, making the patients feel more comfortable and relaxed. The correct answer to each item was recorded by correctly pronouncing the core word, the key word, and the distractor. Each correct answer was scored one point, and incorrect answers or the answer, “I do not know” were scored 0, so the possible results ranged from 0 to 50 [36]. Upon completion of the questionnaire, the examiner summed up the points. A score of 0–41 points indicated an inadequate level of health literacy, and a score of 42–50 points indicated an adequate level of health literacy [35]. The procedure took 8–10 min.

### 2.1. Ethical Issues

The study was performed in accordance with ethical principles and human rights according to medical research standards. The study protocol was approved by the Ethics Committee of the General County Hospital in Požega (no. 02-7/1/1-4-2020). All study patients signed an informed consent form for participation in the study.

### 2.2. Statistical Analysis

Categorical data were expressed by absolute and relative frequencies. Differences between categorical variables were tested by χ^2^ test and Fisher exact test as necessary. The normality of distribution of numerical variables was tested by Shapiro–Wilk test. Numerical data were expressed by median and interquartile range (IQR) for data that did not follow normal distribution. The means of numerical variables of interest were evaluated by 95% confidence interval (95% CI). Differences in numerical variables between two independent groups of patients were tested by Mann–Whitney U test (with Hodges–Lehmann median difference), whereas differences in numerical variables among three or more independent groups were tested by Kruskal–Wallis test (post hoc Conover) [38]. Binary logistic regression analysis was conducted to assess the impact of multiple factors (gender, age, place of residence, level of education, employment status, T2DM, AH, and BMI) on the probability of health literacy.

All *p* values were two-tailed. The level of statistical significance was set at α = 0.05. Statistical data analysis was performed with MedCalc^®^ statistical software version 20.026 (MedCalc Software Ltd., Ostend, Belgium; https://www.medcalc.org; 2021) [39] and SPSS statistical program (IBM Corp., 2017. IBM SPSS Statistics for Windows, IBM Corp, Armonk, NY, USA) [40].

## 3. Results

The study included 500 patients (211 (42.2%) male and 289 (57.8%) female; median age, 63 years (interquartile range, 42–73)).

### 3.1. Morbidity and Nutritional Status of Study Patients

Concerning associated diseases, AH was present in 368 (73.6%) and T2DM in 132 (26.4%) study patients; both diseases were present in 110 (22%), and one of the two in 174 (34.8%) patients (Table 1).

Median patient body weight was 80 kg (range, 43–170), and medio BMI was 27.44 kg/m^2^ (range, 15.99–31.02). According to BMI, there were 140 (28%) normal-weight, 6 (1.2%) underweight, 196 (39.2%) overweight, and 158 (31.4%) obese patients (Table 2).

### 3.2. Health Literacy

**Figure 1:**
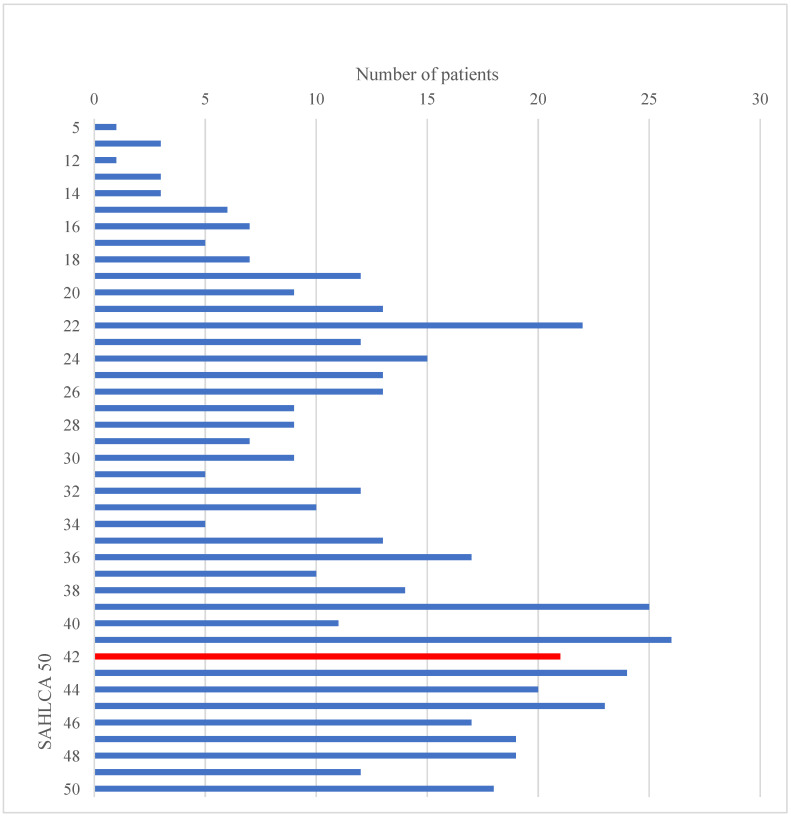
Patient distribution according to SAHLCA-50 questionnaire score: red line–cut-off value discriminating between subjects with an inadequate level of health literacy (0–41 points) and adequate health literacy (42–50 points).

Health literacy was assessed by use of the SAHLCA-50 questionnaire, the Croatian version of the SAHLSA-50 questionnaire, with a score range of 0–50. The median score was 37 (IQR 25–44), ranging from 5 to 50 points. Patient distribution according to the SAHLCA-50 score is illustrated in Figure 1.

### 3.3. Association between Health Literacy and Patient Characteristics

Female patients were found to have a statistically significantly higher level of health literacy than male patients (Mann–Whitney U test, *p* = 0.004). A statistically significantly lower level of health literacy was recorded in patients aged ≥61 as compared with younger age groups (Kruskal–Wallis test, *p* < 0.001).

With respect to level of education, patients with a university degree or higher showed a statistically significantly higher level of health literacy as compared with patients with high school and complete/incomplete elementary school. A higher level of health literacy was also recorded in patients with college education in comparison to those with lower levels of education (Kruskal–Wallis test, *p* < 0.001). Retired patients showed a lower level of health literacy as compared with all other employment categories (Kruskal–Wallis test, *p* < 0.001) (Table 3).

According to the SAHLCA-50 score and the cut-off of 42 points, 173 (34.6%) patients had adequate health literacy, whereas 327 (65.4%) patients showed an inadequate level of health literacy. All differences in the distribution of patient health literacy according to demographic characteristics were statistically significant (χ^2^-test, *p* < 0.001). Of 289 (58%) female patients, 118 (68%) showed adequate health literacy. On the contrary, of 278 (55.6%) male patients aged ≥ 61 years, 228 (69.7%) showed an inadequate level of health literacy. The level of health literacy was higher among patients living in urban areas (*n* = 101; 58%). With respect to level of education, patients with high school and higher levels of education showed a higher level of health literacy as compared with patients with incomplete/completed elementary school. Of 275 (55%) retired patients, a significantly higher proportion showed an inadequate level of health literacy (*n* = 224; 69%) (Table 4).

With respect to morbidity, patients with AH had an inadequate level of health literacy (lower score on SAHLCA-50) as compared to those without AH (32 vs. 40 points; Mann–Whitney U test, *p* < 0.001). Likewise, patients with T2DM scored 31 points versus 39 points in patients without T2DM (Mann–Whitney U test, *p* < 0.001). Patients suffering from both AH and T2DM scored 31 points versus 33 points in those with either AH or T2DM and 41 points in patients with neither AH nor T2DM (Kruskal–Wallis test, *p* < 0.001). There were no statistically significant differences in SAHLCA-50 scores according to the patient nutritional status. Of 262 (52%) AH patients, an inadequate level of health literacy was recorded in 197 (60%) patients (χ^2^-test, *p* < 0.001). In the group of 132 (26%) T2DM patients, the proportion of patients showing an inadequate level of health literacy was significantly greater (*n* = 104; 32%; χ^2^-test, *p* < 0.001). The group of 103 (60%) patients free from either AH or T2DM showed a significantly higher level of health literacy (χ^2^-test, *p* < 0.001). There was no statistically significant difference in health literacy according to patient nutritional status (Table 5).

Regression analysis was used to evaluate the contribution of several factors to the patient’s illiteracy. Bivariate regression analysis showed that an age of 61 and older (odds ratio (OR = 8.17), having completed (OR = 113.3), or not completing elementary school (OR = 550.8) had the strongest influence on health illiteracy, as well as work status vs. retirement (OR = 6.54) (Table 6).

## 4. Discussion

According to the results obtained in this study, only 34.6% of hospitalized patients showed an adequate level of health literacy, which is consistent with the results reported from previous studies on health literacy in other EU member countries [31]. In the present study, a high proportion of hospitalized patients suffered from AH and T2DM, which was expected, considering the high prevalence of these diseases. Although literature data point to the high prevalence of chronic noncommunicable diseases, the population groups at risk are quite likely to be underestimated, with many undiagnosed AH and T2DM cases causing additional concerns [41,42].

Considerably better results were achieved in terms of health literacy by study patients free from AH and T2DM, which could be attributed to the possible contribution of appropriate health literacy in the prevention of chronic, noncommunicable diseases. These results are consistent with those reported in previous international studies [43,44].

The present study revealed a low-level proportion of only 16% of T2DM patients showing an adequate level of health literacy. These results should serve as an indication to health care professionals in planning educational procedures, adjusting interventions, and implementing an individualized approach according to the level of patient health literacy. Efficient T2DM control by patients requires certain knowledge, which in turn requires an adequate level of health literacy. Results of studies on the proportion of T2DM patients with an adequate level of health literacy ranged from 7% in Switzerland to 82% in Taiwan [45].

The level of health literacy was higher among AH patients (38%) as compared with T2DM patients, although still below an adequate level of health literacy, whereas the proportion of patients suffering from both AH and T2DM with an adequate level of health literacy was worryingly low (only 13%).

An inadequate level of health literacy was associated with the development of cardiovascular diseases, which in turn results in inappropriate care for one’s own health and use of health services [46,47]. It should be noted that studies on the association of health literacy and development of T2DM have reported inconsistent results, including nonsignificant association between an inadequate level of health literacy and T2DM occurrence [48,49]. These inconsistent results might be explained by different tools used for health literacy assessment in different studies, as well as by possible geographical and cultural variations among different populations [50].

Previous studies have demonstrated AH and T2DM to have sociodemographic characteristics, anthropometric factors, and lifestyle parameters in common [42,51]. Obesity is a known risk factor for a number of diseases, including other cardiovascular diseases [52], T2DM [53], and AH [54].

The present study identified a high proportion of overweight (39.2%) and obese (31.4%) subjects among hospitalized patients. These results are consistent with the results of a study assessing the prevalence of overweight and obesity, which revealed that the share of these two categories across Europe was highest in Croatia. In 2019, as many as 65% of the adult population in Croatia were overweight [55]. There are several reasons for such a high prevalence of obesity, including modern sedentary lifestyle coupled with inadequate physical activity, poor dietary habits, culturally specific factors, and food culture in the area. 

The results obtained in the present study clearly point to the urgent need for planning public health activities to help promote a healthy lifestyle, along with a reduction in overweight and obesity. Culturally specific acceptable methods need to be designed to achieve the expected results [56].

Many studies have reported on a negative correlation of health literacy and overweight [47,57,58,59,60]. However, the present study revealed a high prevalence of obesity that was not associated with the level of health literacy, as also demonstrated in a study conducted in Iran [61]. Such a discrepancy among study results could be explained by various limitations related to research methodology, e.g., instruments used to measure health literacy. The very concept of health literacy is a complex construct, and measuring procedures are heterogeneous. Therefore, lifestyle, geographical variations, and cultural characteristics are among the factors that may influence the association of health literacy and nutritional status.

In the present study, female patients had significantly higher levels of health literacy, which is similar to findings of other studies [57,62,63]. This finding may be associated with the greater role women traditionally and culturally play in health and healthcare issues for their family members. However, opposite results were reported in a study investigating the association of health literacy with lifestyle modifications and health promotion, wherein an inadequate level of health literacy was recorded in female subjects, which could be attributed to their lower level of education [64].

Literature data point to the association of health literacy with a variety of patient characteristics. In the present study, the level of health literacy increased with each level of education and with employment status; the level of health literacy was highest in employed subjects, which is consistent with other literature reports [65]. This finding appears logical because a higher level of formal education is expected to be accompanied by improved health literacy.

An inadequate level of health literacy was recorded among older adults as compared with younger age groups, which was expected and is consistent with other studies that reporting on inadequate health literacy being more common in elderly patients with poor health condition, which additionally aggravates their health outcome. Therefore, additional educational activities within healthcare services for this population group may prove useful [46]. The results of the present study identified the target population for such activities to raise their level of health literacy. It may be that younger age groups are focused on their health, which is in line with the trend of global increases in individualism described by Santos and colleagues in their research [66]. A study investigating trends and prevalence of healthy lifestyles in young people from 32 countries showed a slight trend of compliance with a healthy lifestyle in this population group [67].

Healthcare workers may occasionally be unaware of the problems originating from an inadequate level of health literacy in their patients [68,69]; therefore, it is necessary is to identify the issues of inadequate health literacy and adjust communication with patients on a comprehensible level. This could be achieved through practical workshops for assessment of health literacy, adjustment of communication skills, checking of understanding the instructions administered, and increasing the number of healthcare workers who can identify and respond to the needs of patients with an inadequate level of health literacy [70,71].

The results of this study indicate that health literacy deficit is a challenge for public health. Actions should address the population at large in order to reduce health literacy variations caused by socioeconomic factors. Reducing health literacy deficits and social gradients in health literacy should be considered a priority in planning strategies, programs, and activities in public health and population health education [72].

Creation of health promotion actions should be substantiated by planning and distribution of due resources, along with a targeted reduction in the burden posed by AH and T2DM in modern society, with simultaneous efficient management of these diseases [73]. Patients suffering from AH and T2DM are faced with the unfavorable fact of their long-lasting condition; therefore, they should be offered continuing education, assistance in understanding their disease, and efficient management and orientation in the healthcare system. A study conducted in Croatia showed that continuing education of T2DM patients led to improved disease management and a reduction in excessive BMI [74].

Healthcare workers should be aware of the variable level of health literacy and adjust their interventions according to the needs and possibilities of each individual patient. Health information should be adjusted to the level of health literacy of each patient to enable him/her to take an active part in the management of disease, leading to taking control over one’s own health in daily life [75].

In spite of some limitations, the results of this study suggest that health literacy may have a major role in the prevalence of chronic diseases. Therefore, the level of health literacy should be upgraded in the population at large in order to promote the effects of health-promoting interventions. The connection between health literacy and health indicators should be investigated in more detail with larger samples covering a larger area through prospective and interventional research designs. 

Documenting the importance of poor patient health literacy in chronic disease programs and comprehending the ways of alleviating its effects are additional research issues to stimulate the reasoning of how changes in the healthcare system can favorably influence the obstacles associated with low levels of health literacy [76]. Further research should be focused on longitudinal studies to identify the causes, outcomes, and consequences, as well as the potential impact of health condition on changes in health literacy [77]. The high prevalence of low levels of health literacy is a serious problem in our society that requires a multimodal approach for resolution. Additional efforts should be invested to upgrade the level of health literacy in order to reduce health inequality in society based on the concept of health literacy [78].

## 5. Conclusions

The present study demonstrates a significant association of inadequate health literacy with AH, and T2DM but not with obesity. Male gender, low level of education, rural place of residence, retirement, and advanced age were found to be significant predictors of an inadequate level of health literacy. Actions aimed at upgrading health literacy might help to prevent chronic, noncommunicable diseases, such as AH and T2DM.

## Figures and Tables

**Table 1:** Patient distribution according to associated diseases (*n* = 500).

Associated Disease	*n* (%)
Arterial hypertension	262 (52.4)
T2DM	132 (26.4)
Arterial hypertension and T2DM	110 (22)
Either arterial hypertension or T2DM	174 (34.8)
Neither arterial hypertension nor T2DM	216 (43.2)

**Table 2:** Mean body weight, body height, body mass index (BMI), and patient distribution according to nutritional status (*n* = 500).

Characteristic	Median (Interquartile Range)
Body weight (kg)	80 (70–90)
Body height (cm)	170 (163–175)
Body mass index (BMI) (kg/m^2^)	27.44 (24.45–31.02)
Nutritional status:	*n* (%)
Underweight (BMI ≤ 18.5 kg/m^2^)	6 (1.2)
Normal weight (18.5 ≤ BMI ≤ 24.9 kg/m^2^)	140 (28)
Overweight (25.0 ≤ BMI ≤ 29.9 kg/m^2^)	196 (39.2)
Obesity I (30.0 ≤ BMI ≤ 34.9 kg/m^2^)	108 (21.6)
Obesity II (35.0 ≤ BMI ≤ 39.9 kg/m^2^)	26 (5.2)
Obesity III (BMI ≥ 40.0 kg/m^2^)	24 (4.8)

**Table 3:** Differences in SAHLCA-50 score according to patient characteristics (*n* = 500).

Characteristic	Median (Interquartile Range) SAHLCA-50		*p*
Gender:			
Male	36 (25–42)	^†^ Difference = 3 95% CI = 1–4	0.004 *
Female	39 (26–5)		
Age group (years):			
<30	44 (39–47)	H test = 123.4 df = 4	<0.001 ^‡ §^
31–40	44 (39–48)		
41–50	43 (34–46)		
51–60	41 (36–44)		
≥61	28 (22–39)		
Level of education:			
Incomplete elementary school	22 (18–26)	H test = 246.1 df = 4	<0.001 ^‡ ††^
Elementary school	26 (22–34)		
High school	41 (36–45)		
College	45 (41–48)		
University degree or higher	47 (44–49)		
Employment status:			
Employed	43 (38–47)	H test = 113.3 df = 3	<0.001 ^‡ §§^
Unemployed	41 (33–45)		
Occasionally employed	43 (33–45)		
Retired	28 (22–39)		

CI, confidence interval; * Mann–Whitney U test; ^†^ Hodges–Lehmann median difference; ^‡^ Kruskal–Wallis test (post hoc Conover); ^§^ significant difference at *p* < 0.05 (≥61 age group vs. all other age groups; <30 age group vs. 51–60 age group); ^††^ significant difference at *p* < 0.05: university and higher degree vs. high school, completed elementary school, incomplete elementary school; college vs. high school, completed elementary school, incomplete elementary school; ^§§^ significant difference at *p* < 0.05 between retired patients and employed/unemployed patients.

**Table 4:** SAHLCA-50 score according to demographic characteristics (*n* = 500).

Characteristic	*n* (%)			*p* *
	Adequate Health Literacy (*n* = 173) (42–50 points)	Inadequate Health Literacy (*n* = 327) (0–41 points)	Total	
Gender:				
Male	55 (26)	156 (74)	211 (42)	0.001
Female	118 (41)	171 (59)	289 (58)	
Age group (years):				
<30	43 (64)	24 (36)	67 (13)	<0.001
31–40	34 (63)	20 (37)	54 (11)	
41–450	21 (60)	14 (40)	35 (7)	
51–460	25 (38)	41 (62)	66 (13)	
≥61	50 (18)	228 (82)	278 (56)	
Place of residence:				
Rural	72 (25)	221 (75)	293 (59)	<0.001
Urban	101 (49)	106 (51)	207 (41)	
Level of education:				
Incomplete elementary school	1 (1)	81 (99)	82 (16)	<0.001
Elementary school	6 (6)	100 (94)	106 (21)	
High school	111 (45)	133 (55)	244 (49)	
College	21 (72)	8 (28)	29 (6)	
University and higher	34 (87)	5 (13)	39 (8)	
Employment status:				
Employed	82 (60)	55 (40)	137 (27)	<0.001
Unemployed	37 (44)	47 (56)	84 (17)	
Occasionally employed	3 (75)	1 (25)	4 (1)	
Retired	51 (19)	224 (81)	275 (55)	

* χ^2^-test.

**Table 5:** Health literacy (SAHLCA-50 score) according to patient characteristic (*n* = 500).

Characteristic	*n* (%)			*p* *
	Adequate Health Literacy (42–50 points)	Inadequate Health Literacy (0–41 points)	Total	
**Arterial hypertension (AH):**				
Yes	108 (45)	130 (55)	238 (48)	<0.001
No	65 (25)	197 (75)	262 (52)	
Type 2 diabetes mellitus (T2DM):				
No	145 (39)	223 (61)	368 (74)	<0.001
Yes	28 (21)	104 (79)	132 (26)	
AH/T2DM:				
Both	23 (21)	87 (79)	110 (22)	<0.001
Either AH or T2DM	47 (27)	127 (73)	174 (35)	
Neither	103 (48)	113 (52)	216 (43)	
Nutritional status:				
Underweight	2 (33)	4 (67)	6 (1)	0.7 ^†^
Normal weight	51 (36)	89 (64)	140 (28)	
Overweight	71 (36)	125 (64)	196 (39)	
Obesity	49 (31)	109 (69)	158 (32)	
Total	173 (100)	327 (100)	500 (100)	

* χ^2^-test; ^†^ Fisher exact test.

**Table 6:** Predicting the probability of an inadequate levels of health literacy (bivariate regression analysis).

Characteristic	ß	Wald	*p*	OR	95% Cl
Gender (M)	0.67	11.59	<0.001	1.96	1.33–2.88
Age group (years):					
31–40	0.05	0.02	0.89	1.05	0.50–2.22
41–50	0.18	0.17	0.68	1.19	0.52–2.77
51–60	1.08	8.98	0.003	2.94	1.45–5.95
≥61	2.10	49.4	<0.001	8.17	4.55–14.68
Place of residence (city)	−1.07	30.5	<0.001	0.34	0.23–0.50
Level of education:					
College education	0.95	2.25	0.13	2.59	0.75–8.97
High school education	2.09	17.89	<0.001	8.15	3.08–21.5
Elementary school	4.73	55.10	<0.001	113.3	32.5–395.18
Incomplete elementary school	6.31	32.07	<0.001	550.8	62.01–4892.6
Employment status:					
Unemployed	0.64	5.18	0.02	1.89	1.09–3.28
Part-time employee	−0.69	0.36	0.55	0.49	0.05–4.90
Retired	1.87	64.9	<0.001	6.54	4.14–10.34
Arterial hypertension (yes)	0.92	22.8	<0.001	2.52	1.72–3.67
Type 2 diabetes (yes)	0.88	13.7	<0.001	2.42	1.51–3.85
AH/T2DM					
Both diseases	1.24	20.8	<0.001	3.45	2.03–5.87
Pne of the diseases	0.90	17.0	<0.001	2.46	1.61–3.78
Nutrition (obese)					
Underweight	0.13	0.02	0.88	1.14	0.20–6.36
Normal weight	−0.01	0.001	0.97	0.99	0.63–1.56
Obesity	0.23	1.06	0.30	1.26	0.81–1.97

ß–regression coefficient; OR—odds ratio; CI—confidence interval.

## Data Availability

The data presented in this study are available on request from the corresponding author.
